# Docosahexaenoic Acid in Combination with Dietary Energy Restriction for Reducing the Risk of Obesity Related Breast Cancer

**DOI:** 10.3390/ijms19010028

**Published:** 2017-12-22

**Authors:** Andrea Manni, Karam El-Bayoumy, Henry Thompson

**Affiliations:** 1 Milton S. Hershey Medical Center, Departments of Medicine, Penn State University College of Medicine, 500 University Drive, Hershey, PA 17033, USA; 2 Milton S. Hershey Medical Center, Departments of Biochemistry and Molecular Biology, Penn State University College of Medicine, 500 University Drive, Hershey, PA 17033, USA; kee2@psu.edu; 3 Cancer Prevention Laboratory, Colorado State University, 221 Shepardson Building, 1173 Campus Delivery, Fort Collins, CO 80523-1173, USA; henry.thompson@colostate.edu

**Keywords:** obesity-related breast cancer, docosohexaenoic acid (DHA), dietary energy restriction (DER), combination of DHA and DER for breast cancer risk reduction, breast density, stearoyl-coA-desaturase (SCD-1)

## Abstract

There is strong evidence that obesity poses a significant risk factor for postmenopausal breast cancer. There are multiple mechanisms by which obesity can predispose to breast cancer, prominent among which is the creation of a pro-inflammatory milieu systemically in the visceral and subcutaneous tissue, as well as locally in the breast. Although dietary intervention studies have shown in general a favorable effect on biomarkers of breast cancer risk, it is still unclear whether losing excess weight will lower the risk. In this manuscript, we will review the evidence that omega-3 fatty acids, and among them docosahexaenoic acid (DHA) in particular, may reduce the risk of obesity related breast cancer primarily because of their pleotropic effects which target many of the systemic and local oncogenic pathways activated by excess weight. We will also review the evidence indicating that intentional weight loss (IWL) induced by dietary energy restriction (DER) will augment the tumor protective effect of DHA because of its complementary mechanisms of action and its ability to reverse the obesity-induced alterations in fatty acid metabolism predisposing to carcinogenesis. We believe that the combination of DER and DHA is a promising safe and effective intervention for reducing obesity-related breast cancer risk which needs to be validated in appropriately designed prospective, randomized clinical trials.

## 1. Introduction

Investigative studies in clinical trials over the past two decades have improved recurrence free and overall survival rates of breast cancer patients by focusing on early diagnosis and improved treatment strategies. However, patients and their physicians have increasingly recognized the substantial cost and emotional burden resulting from the diagnosis of breast cancer and its treatment. Even if the tumor is diagnosed at an early stage, women must undergo surgery followed usually by radiotherapy and adjuvant chemo or endocrine therapy. These considerations highlight the importance of breast cancer prevention as the optimal method to reduce breast cancer morbidity and mortality. The two selective estrogen receptor modulators, Tamoxifen and Raloxifene, have been shown to be effective agents in reducing the incidence of estrogen receptor positive breast cancer by 50% and 38% respectively [1,2]. However, neither drug is able to reduce the incidence of estrogen receptor negative tumors which are more aggressive and associated with a shorter survival compared to estrogen receptor positive cancers. Furthermore, the acceptance of Tamoxifen and Raloxifene even by women at high risk of breast cancer has been shown to be poor primarily because of fear of side effects such as thromboembolic events which, although rare, are significant when considering that these drugs are given to healthy women [3].

We believe that two major challenges need to be met in order to make significant progress in breast cancer prevention. First, a multi-targeted approach is needed employing interventions with complementary mechanism of action since multiple cellular pathways, in addition to the estrogen receptor, contribute to the development of breast cancer. In addition, this intervention needs to be safe and preferably health-promoting in order to be acceptable by the population of healthy women at large for breast cancer prevention. We believe that n-3FA (omega-3 fatty acids) meet both challenges because of their pleotropic mechanism of action and their safety profile. In this manuscript, we will review the evidence to indicate that n-3FA and docosohexaenoic acid (DHA) in particular maybe particularly effective in preventing obesity related breast cancer, a highly prevalent phenotype due to the obesity epidemic in western societies. We will also review the evidence to suggest that IWL secondary to dietary energy restriction (DER) will potentiate the tumor protective effect of n-3FA and specifically DHA.

## 2. Obesity and Breast Cancer

Excess body weight is reaching epidemic proportions with 65% of adults in the United States being either overweight (body mass index (BMI) 25.0–29.9 kg/m^2^) or obese (BMI ≥ 30 kg/m^2^) [4]. After smoking, obesity is now considered to be the most important modifiable cause of cancer as it has been associated with increased risk of many cancers including postmenopausal breast cancer [5,6]. Although obesity in postmenopausal women has been primarily associated with estrogen receptor positive tumors, an increased incidence of estrogen receptor negative tumors has also been reported in both pre- and postmenopausal obese women compared to lean subjects [7,8]. In addition, obesity has been associated with worse prognosis, including increased mortality in women diagnosed with breast cancer [9]. There are multiple mechanisms by which obesity predisposes to breast cancer such as altering the production and bioavailability of critical mitogens such as insulin growth factor-1 [10]. Obesity is also associated with a state of insulin resistance [11] which can contribute to breast cancer development as a result of the high circulating level of insulin acting as a growth factor [12]. In addition, obesity induces changes in circulating adipokines such as leptin [13] and adiponectin [14] which are protumorigenic. Leptin plasma levels are increased in obese subjects and higher leptin levels are associated with an increase in breast cancer risk [15]. Conversely, adiponectin levels are reduced in obesity and in inverse association between serum adiponectin and breast cancer risk has been observed [16]. The creation of a proinflammatory milieu systemically in the visceral and subcutaneous fat [17,18] and locally in the breast [19] has been recognized as a major mechanism by which obesity promotes the development of breast cancer. Typical inflammatory foci called crown-like structure have been identified in the visceral fat [20,21] and mammary glands [17,19] which are characterized by necrotic adipocytes encircled by CD68+ proinflammatory macrophages. Saturated fatty acids released by the adipocyte stimulate NFκB activity in the macrophages leading to increased levels of inflammatory cytokines such as tumor necrosis factor α (TNFα), interleukin-1β (IL-1β) and prostaglandin E_2_ (PGE_2_) [17]. PGE_2_ has been shown to stimulate the cyclic AMP/PKA signal transduction pathway which activates cytochromeP450 (CYP)19 transcription resulting in increased aromatase expression and estrogen production [22]. These findings highlight the link between obesity, inflammation and the creation of a hyperestrogenic milieu promoting the development of hormone responsive postmenopausal breast cancer. It is also recognized that a state of inflammation frequently associated with obesity may also be present in a subset of lean subjects while it may be absent in some obese women [23]. These findings indicate that biomarkers of inflammation such as insulin resistance and the presence of crown-like structure may be better predictors of breast cancer risk than an elevated BMI alone [22,23].

Obesity has also been shown to alter fatty acid metabolism in a way that may predispose to mammary carcinogenesis. Bettaieb et al. [24] reported that overweight individuals exhibit increased adipose tissue expression of sEH (soluble epoxide hydrolase) a cytosolic enzyme which hydrolyzes and inactivates epoxygenated oxylipins produced by CYP epoxygenases. Oxylipins generated from DHA through this pathway, EDP have been shown to have potent antitumor actions at multiple levels including angiogenesis, growth, and metastasis [25]. These findings highlight the importance of the CYP pathway of lipid metabolism and n-3FA in particular with regard to antitumor activity which has received limited attention in the literature [26].

Although the association between obesity and breast cancer is well established, it is unclear whether losing excess weight will lower the risk [27]. The lack of randomized clinical trials precludes us from establishing a causal relationship between weight loss and a reduction in breast cancer risk. The best evidence in support of a weight loss induced reduction of postmenopausal breast cancer risk comes from bariatric surgery studies, although most have been observational in design [28,29,30]. Of interest, a recent paper [31] reported a reduction in breast density, a validated biomarker of breast cancer risk [32,33,34], in postmenopausal obese women following weight loss induced by bariatric surgery. These findings, however, may not be applicable to the obese population at large since they examined the effects of extreme and rapid amounts of weight loss resulting from the procedure. Dietary energy restriction studies, usually of short duration, have shown in general a favorable effect on circulating biomarkers of breast cancer risk [35]. A recent 12-month dietary intervention trial leading to an 8.5% weight reduction in overweight and obese postmenopausal women significantly reduced circulating levels of some markers of oxidative stress [36] which may be involved in tumor initiation and promotion [37,38]. In a six-month study conducted in overweight and obese postmenopausal women at increased risk for breast cancer, Fabian, C.J. et al. [39] reported that a behavioral-induced weight loss of at least 10% resulted in favorable modulation of many putative serum and tissue biomarkers of breast cancer risk.

In the aggregate, these studies [35,36,39] suggest that even relatively modest weight reductions (~10%) achievable with behavioral interventions may have a protective effect in overweight and obese subjects. Future randomized clinical trials involving a larger number of women at risk should be conducted to determine the impact of weight loss on breast cancer prevention using relevant biomarkers of breast cancer risk.

### 2.1. Omega-3 Fatty Acids and Obesity Related Breast Cancer Prevention

The influence of the fatty acid composition of the diet to breast cancer development has been extensively investigated in the literature. Among the fatty acids, n-3FA and n-6FA have been suggested to decrease and increase breast cancer risk respectively [40]. Preclinical experiments conducted over the last three decades in numerous experimental systems have been in general supportive of a cause-effect relationship between intake of n-3FA and inhibition of mammary carcinogenesis, although the results have been variable [41]. It should be noted that the vast majority of these studies were conducted in normal weight rodents. Very few studies (see below) have investigated the protective effects of n-3FA in obesogenic models. Epidemiological studies have yielded conflicting results regarding the protective effect of n-3FA against breast cancer [42]. Of interest and relevant to the topic of this review, in a case control study involving Mexican women, increased n-3FA intake was associated with decreased risk of breast cancer for obese women (OR 0.58, 95% CI 0.39–0.87, *p* = 0.008) [43]. A recent metaanalysis of data from 21 independent prospective cohort studies revealed that dietary intake of marine n-3FA was associated with a 14% reduction in breast cancer risk [44]. Importantly, a dose-response effect was noted with a 5% lower risk of breast cancer per 0.1 g/day increment of n-3FA intake [44]. We believe that at least one of the variables accounting for the inconclusive results of the epidemiological studies is the heterogeneity of the subject populations under investigation. Our review of the literature as well as our own data suggests that n-3FA may be preferentially effective in reducing breast cancer risk in obese women.

From a mechanistic point of view, n-3FA inhibit several of the oncogenic pathways activated by obesity as summarized by us in a recent review [45]. Preclinical studies have suggested that n-3FA reduce obesity related inflammation and insulin resistance [46,47]. In addition, fish oil rich in n-3FA has been shown to increase the plasma level of adiponectin in rodents and in human subjects and to decrease plasma leptin concentrations [48] thus reversing the protumorigenic adipokine profile induced by obesity. A major mechanism by which n-3FA reduce the risk of obesity related breast cancer is through suppression of inflammation [42,49]. Our data, obtained in a preclinical model of mammary carcinogenesis, indicate that n-3FA induce PPARγ (peroxisome proliferator-activated receptor γ) and downregulate NFkβ [50]; both of these effects would be expected to result in inhibition of PGE_2_ and consequently estrogen production. It has also been suggested that a high intake of n-3FA relative to n-6FA may decrease endogenous estrogen production via inhibition of aromatase activity/expression [51]. In support of this contention, pioglitazone, a recognized inducer of PPARγ transcriptional activation, has indeed been reported to inhibit aromatase induction by PGE_2_ [52]. Particularly supportive of a preferential protective effect of n-3FA against obesity-related breast cancer is a report by Ford, N.A. et al. [53]. Using two mouse models of postmenopausal triple negative breast cancer (basal-like and claudin low), these investigators found that the combination of EPA (eicosapentaenoic acid) and DHA exerted an antitumor effect only in obese mice. In addition, this treatment blocked many of the protumorigenic effects of obesity.

### 2.2. DHA, Breast Density, and Obesity Related Breast Cancer Risk

**Figure 1:**
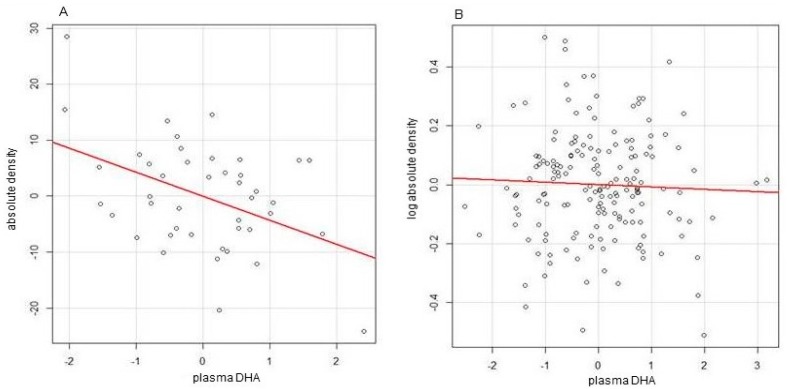
Partial regression plot of the dependence of absolute breast density on docosahexaenoic acid (DHA) after adjusting for other predictors for body mass index (BMI) > 29 (**A**) and ≤29 (**B**) Reproduced with permission from [54].

Support for a preferential protective effect of n-3FA against obesity related breast cancer is provided by the results of our recently published clinical trial [54]. In this study, in addition to testing the individual and combined effects of the FDA-approved formulation of n-3FA Lovaza (a combination of EPA and DHA) and the antiestrogen Raloxifene in reducing breast density, a validated biomarker of breast cancer risk [32,33,34], we tested the hypothesis that BMI influences the relation between breast density and n-3FA. Using a multivariate linear regression analysis [55], we observed a regression coefficient of absolute breast density on DHA of −4.301 (*p* = 0.0076) for the dataset of BMI above 29 (Figure 1A) whereas the regression coefficient was 0.0080 (*p* = 0.59) for the dataset of BMI ≤ 29 (Figure 1B). No correlation between breast density and EPA was found in either dataset. This indicates that obese women may preferentially experience breast cancer risk reduction from n-3FA administration and support the rationale for targeting this sub-population of high-risk subjects in future clinical trials focusing on testing the protective effect of DHA as the n-3FA of choice for reduction of breast cancer risk. DHA has also been found to be superior to EPA as an antitumor agent in experimental breast cancer models [56,57].

**Figure 2:**
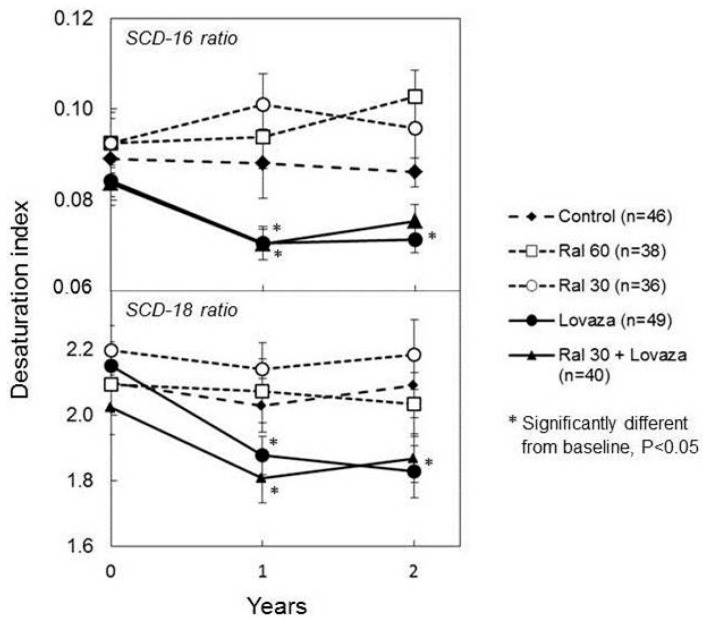
Individual and combined effects of Lovaza and Raloxifene on desaturation index, an indicator of stearoyl-coA-desaturase (SCD-1) activity. Data represent means ± SEM. Reproduced with permission from [63].

**Figure 3:**
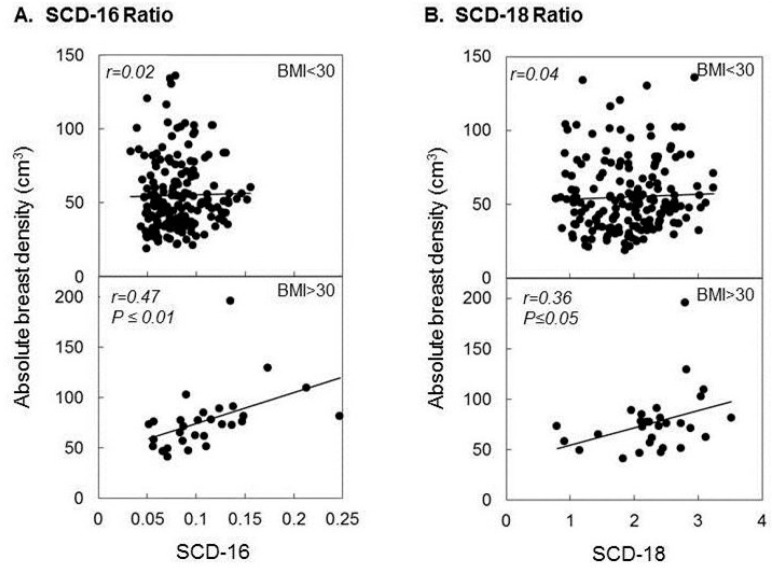
Correlation between SCD-1 activity, expressed as either SCD-16 ratio (**A**) or SCD-18 ratio (**B**) and absolute breast density in women with BMI < 30 (upper panels) or >30 (lower panels). Reproduced with permission from [63].

Our preclinical data indicate that inhibition of saturated fatty acids (SFA) synthesis is a major mechanism of antitumor action of n-3FA [50]. Therefore, we became interested in determining whether changes in one of the lipogenic parameters could be predictive of the observed inverse correlation between plasma DHA level and breast density observed in our clinical trial [54]. Preclinical and epidemiological data indicate that activation of stearoyl-coA-desaturase (SCD-1), a Δ9 fatty desaturase responsible for the conversion of SFA to Monounsaturated fatty acids (MUFA) is potentially a critical factor both in the development of obesity [58] and cancer including breast cancer [59]. Epidemiological studies have indeed shown a positive correlation between high levels of SCD-1 expression and the risk of breast cancer [60,61] as well as adiposity [62]. However, no data are available in humans about the regulation of this enzyme by chemopreventive agents and it is not known how changes in SCD-1 activity relate to established biomarkers of breast cancer risk. To fill this gap, we measured SCD-1 activity in stored plasma samples of subjects enrolled in our clinical trial [63]. We observed that administration of Lovaza but not Raloxifene significantly reduced SCD-1 in postmenopausal women at increased risk of breast cancer based on high breast density (Figure 2). Importantly, we showed that decreasing levels of SCD-1 were associated with a progressive reduction in breast density but only in obese women (Figure 3, lower panels). In contrast, no association was found between SCD-1 and breast density in women with BMI < 30 (Figure 3, upper panels). These results, in conjunction with those of our clinical trial, suggest that downregulation of SCD-1 by n-3FA and DHA in particular may not be tumor protective in non-obese women (at least as determined by a reduction in breast density) but may be selectively protective in obese subjects where decreasing levels of SCD-1 were associated with a progressive decrease in absolute breast density. This observation again reinforces our hypothesis that obese women may preferentially benefit from the antitumor action of n-3FA and DHA in particular.

### 2.3. The Combination of n-3FA and DER for Optimal Breast Cancer Prevention

There is considerable evidence in the literature to suggest that the combination of n-3FA and DER may lead to optimal inhibition of obesity related breast cancer which is characterized by a high degree of inflammation. As we discussed above, the anti-inflammatory and anti-oxidative actions of n-3FA are well established [42,49]. Calorie restriction has also been shown to have potent anti-inflammatory and anti-oxidative effects. Using Fat-1 transgenic mice (Fat-1) that are able to convert n-6FA to n-3FA endogenously, Raman et al. [64] have shown that there is a synergistic effect of endogenously synthesized n-3FA and 40% calorie restriction in lowering proinflammatory cytokines and enhancing the anti-oxidant enzymes. Along the same line, Flachs, P. et al. [65] showed that dietary n-3FA and mild DER in obese mice synergistically reduced the degree of inflammation of the white adipose tissue by synergistic induction of mitochondrial oxidative capacity, lipid catabolism, and specific anti-inflammatory lipid mediators. Our preclinical data have revealed a striking complementarity by which dietary energy restriction and n-3FA affect signaling pathways involved in breast cancer development. They both influence a host of systemic factors (adipokines and insulin/IGF-1) and cell autonomous pathways (AKT-mTOR and lipid metabolism) ultimately leading to inhibition of cell proliferation and induction of apoptotic cell death. However, while the dominant effect of DER is on inducing a proapoptotic environment, primarily via enhancement of the intrinsic pathway [66], the dominant mechanism of antitumor action by n-3FA is by reducing cell proliferation via selective activation of PPARγ and inhibition of lipogenesis [50]. Furthermore, the rationale for combining DER specifically with the n-3FA DHA is strengthened by the finding that obesity may diminish the antitumor effect of DHA against breast cancer by increasing sEH which hydrolyzes and inactivates tumor protective DHA-derived oxylipins produced by CYP epoxygenases [24]. Therefore, DER may potentiate the antitumor action of DHA by reversing the effects of obesity on sEH and thus restoring the levels of these tumor protective compounds.

## 3. Conclusions and Future Directions

Based on the evidence discussed above, the combination of n-3FA and DER offers the promise of being a health-promoting and effective approach to inhibiting the development of obesity related breast cancer. The effectiveness of this intervention in the clinical setting will need to be tested in appropriately designed clinical trials using validated biomarkers of breast cancer risk. Our preclinical data generated in the MNU mammary tumor model showed that a reduction in breast density predicted the antitumor effect of an n-3FA rich diet [67]. Importantly, our clinical data showing an inverse relationship between DHA and absolute breast density selectively in obese women strongly supports the choice of this biomarker as the primary endpoint for future clinical trials [54]. A recent clinical trial has indeed indicated that breast density is a valid biomarker to assess risk reduction induced by weight loss following bariatric surgery in obese postmenopausal women [31]. We realize that the relationship between BMI and breast density is controversial in the literature [68,69]. This may depend to a significant extent upon whether breast density is expressed as percent or absolute density. We observed that absolute density was positively associated whereas percent density was negatively related with BMI in postmenopausal women [70]. This is attributable to “diluting” effect of non-dense breast volume which we found to be positively associated with overall body adiposity [70]. Therefore, a reduction in body weight might be expected to result in an increase in percent breast density. Preclinical data [56,57] and the results of our clinical trial [54] indicate that DHA (as opposed to EPA) is the most active n-3FA against breast cancer and should be preferentially tested in future trials. A largely unexplored topic deserving further investigation is the role of DHA metabolism in mediating its antitumor action. Metabolites of DHA generated through the cyclooxygenase, lipoxygenase, and cytochrome p450 pathways have been shown to have antitumor actions possibly superior to that of DHA itself [25,71]. We have recently reported that one of the lipoxygenase metabolites of DHA, 4-OXO-DHA, was more effective than the parent compound in inhibiting the growth of triple negative breast cancer cells in culture [72]. Furthermore, the production of DHA metabolites is likely to be highly variable among individuals since it is regulated both by genetic [73] and by environmental factors such as obesity [24].

In conclusion, we believe that the inconclusive results reported in the literature over the last three decades on the potential protective effect of fish oil against breast cancer is due to a large extent to the heterogeneity of the fish oil preparations as well as the subject populations under study. We believe that the fish oil breast cancer conundrum can best be resolved by a personalized approach in future clinical trials including the selection of an appropriately targeted population (such as obese subjects) and using a well-defined n-3FA such as DHA based on experimental evidence.

## Abbreviations

n-3FA	omega-3 fatty acids
DHA	docohexaenoic acid
DER	dietary energy restriction
IWL	intentional weight loss
PGE_2_	prostaglandin E_2_
sEH	soluble epoxide hydrolase
CYP	cytochrome p450
EDP	epoxydocoapentaenoic acid
SCD-1	Stearoyl-CoA-desaturase
SFA	saturated fatty acids
MUFA	Monounsaturated fatty acids
